# Entomotoxic Activity of the Extracts from the Fungus, *Alternaria tenuissima* and Its Major Metabolite, Tenuazonic Acid

**DOI:** 10.3390/jof7090774

**Published:** 2021-09-18

**Authors:** Dilara Salimova, Anna Dalinova, Vsevolod Dubovik, Igor Senderskiy, Elena Stepanycheva, Oksana Tomilova, Qiongbo Hu, Alexander Berestetskiy

**Affiliations:** 1 Department of Phytotoxicology and Biotechnology, All-Russian Institute of Plant Protection, Podbelskogo Shosse, 3, Pushkin, 196608 Saint-Petersburg, Russia; d.salimova@vizr.spb.ru (D.S.); adalinova@vizr.spb.ru (A.D.); vdubovik@vizr.spb.ru (V.D.); senderskiy@mail.ru (I.S.); estepanycheva@yandex.ru (E.S.); 2 Institute of Systematics and Ecology of Animals SB RAS, Frunze Str. 11, 630091 Novosibirsk, Russia; toksina@mail.ru; 3 Key Laboratory of Bio-Pesticide Innovation and Application of Guangdong Province, College of Plant Protection, South China Agricultural University, Guangzhou 510642, China; hqbscau@scau.edu.cn

**Keywords:** *Alternaria tenuissima*, extract, bioassays, PCA, tenuazonic acid, *Galleria mellonella*, *Zophobas morio*, *Acheta domesticus*, *Tetranychus urticae*, *Schizaphis graminum*, Sf9

## Abstract

The study of fungal antibiotics in their competitive interactions with arthropods may lead to the development of novel biorational insecticides. Extracts of *Alternaria tenuissima* MFP253011 obtained using various methods showed a wide range of biological activities, including entomotoxic properties. Analysis of their composition and bioactivity allowed us to reveal several known mycotoxins and unidentified compounds that may be involved in the entomotoxic activity of the extracts. Among them, tenuazonic acid (TeA), which was the major component of the *A. tenuissima* extracts, was found the most likely to have larvicidal activity against *Galleria mellonella*. In the intrahaemocoel injection bioassay, TeA was toxic to *G. mellonella* and of *Zophobas morio* with an LT_50_ of 6 and 2 days, respectively, at the level of 50 µg/larva. Administered orally, TeA inhibited the growth of *G. mellonella* larvae and caused mortality of *Acheta domesticus* adults (LT_50_ 7 days) at a concentration of 250 µg/g of feed. TeA showed weak contact intestinal activity against the two phytophages, *Tetranychus urticae* and *Schizaphis graminum*, causing 15% and 27% mortality at a concentration of 1 mg/mL, respectively. TeA was cytotoxic to the Sf9 cell line (IC_50_ 25 µg/mL). Thus, model insects such as *G. mellonella* could be used for further toxicological characterization of TeA.

## 1. Introduction

Herbivores and phytopathogens inhabit the same ecological niches and use plants as a food source and, therefore, can compete for substrates. In this regard, some phytopathogenic microorganisms are assumed to produce metabolites that directly (because of entomotoxic or repellent action) or indirectly (through induced plant responses, suppressed immunity, and the symbiotic microbiota of insects) affect the fitness of arthropods. The study of the antagonistic effect of phytopathogenic microorganisms on the viability, development, and fertility of arthropods is important for the development of methods to control phytophagous insects and vectors of human and animal diseases [1].

Among phytopathogenic fungi, *Alternaria* species have the potential for the production of entomotoxic secondary metabolites. For instance, extracts of approximately 20% of tested isolates of nine *Alternaria* species showed aphicidal activity against the vetch aphid (*Megoura viciae*). The aphid was most sensitive to organic extracts from cultures of *A. saponariae*, *A. japonica*, *A. penicillata*, *A. papavericola*, and *A. tenuissima* [2]. An ethanolic extract from the mycelium of *A. papavericola* (*Brachycladium papaveris*) caused mortality of *M. viciae* comparable to the botanical insecticide NeemAzal [3]. Extracts from *A. alternata* cultures inhibited acetylcholinesterase and exhibited insecticidal and immunosuppressive activity against the cotton leafworm (*Spodoptera litura*) [4,5]. Extracts from *A. destruens* cultures that inhibited the activity of *α*-glucosidase were toxic to *S. litura* with an LD_50_ of approximately 2 mg/g feed [6].

A number of entomotoxic compounds had been purified from cultures of several *Alternaria* species. *A. brassicae* was found to produce depsipeptide phytotoxins, some of which (for example, destruxin B) demonstrate insecticidal properties [7,8]. Methyl-3,8-dihydroxy-6-methyl-4-chloro-9-oxo-9H-xanthene-1-carboxylate and chloromonilinic acid B isolated from *A. sonchi* strongly affected (75% mortality) the common wheat aphid (*Schizaphis graminum*) at a concentration of 1 mg/mL [9]. Altenuene produced by *A. alternata* led to 70% mortality of *S. litura* larvae at 5 mg/g feed [10]. Tenuazonic acid produced by some *Alternaria* species was toxic for the first instar larvae of the common green bottle fly (*Lucilia sericata*) (LD_50_ 120 μg/mL) [11].

Small-spored *Alternaria* spp. may be of special interest as possible producers of entomotoxic metabolites. These species have often been isolated from cadavers or hibernating stages of arthropods. They also demonstrate entomopathogenic properties [12,13,14]. In particular, the strains of the fungus preliminarily identified as *A. infectoria* infected eggs, larvae, and adults of the fig wax scale (*Ceroplastes rusci*) [15]. Interestingly, *A. alternata* strains that were pathogenic to various aphid species did not infect insects from other orders, suggesting the production of aphid-specific toxins by the fungus [16,17]. At the same time, small-spored *Alternaria* spp. of the section *Alternaria* (*A. alternata*, *A. arborescens*, *A. gaisen*, and *A. tenuissima*), which are widespread on various plant substrates as saprotrophs and are weak pathogens [18], can indirectly affect various arthropods through the production of phytotoxins or/and plant immunomodulators [19,20]. Therefore, although the small-spored *Alternaria* spp. are capable of infecting arthropods, the entomotoxic properties of their secondary metabolites are still poorly understood.

The earlier characterized strain MFP253011 of *A. tenuissima* [21] was chosen as the object of this study. The aim of this work was to reveal and characterize the determinants of *A. tenuissima* MFP253011 entomotoxicity. For this purpose, (1) the fungal cultures were grown on five liquid and solid substrates to extract the metabolites with two solvents and then to evaluate the spectrum of their bioactivity; (2) the composition of the extracts was analyzed in relation to the bioactivity to reveal entomotoxicity determinants and their possible indirect effects; and (3) tenuazonic acid (TeA), which was found to be a main component of extracts showing anti-insectan properties, was assayed against various species of model (*Galleria mellonella*) and feed (*Zophobas morio* and *Acheta domesticus*) insects as well as phytophages (*Schizaphis graminum* and *Tetranychus urticae*).

## 2. Materials and Methods

### 2.1. Fungal Strain and Fermentation

The strain MFP253011, gifted by Dr. Philipp Gannibal (Laboratory of Mycology and Phytopathology at the All-Russian Institute of Plant Protection, Saint Petersburg, Russia) and identified as *A. tenuissima* (Kunze) Wiltshire [21], was used in this study. The fungal culture was preserved on standard potato-dextrose agar (PDA) at a temperature of 5 °C. To obtain extracts, the fungus was grown in 1 L Erlenmeyer flasks with 300 mL of a liquid medium. Four standard liquid synthetic and semisynthetic nutrient media that varied by carbon and nitrogen sources were used: modified Czapek media (MCM), M1D, YMG, and Sabouraud media (SAB). The composition (g per L of deionized water, pH 6) was as follows: MCM (glucose—20, NaNO_3_—2, KH_2_PO_4_—1, MgSO_4_—0.5, KCl—0.5, thiamine 1 × 10^−4^, biotin—5 × 10^−6^), M1D (sucrose—45, ammonium tartrate—7.5, MgSO_4_ × 7 H_2_O—5.25, Ca(NO_3_)_2_—0.45, KNO_3_—0.15, KCl—0.15, NaH_2_PO_4_—0.03, FeCl_3_ × 6 H_2_O—0.003, ZnSO_4_ × 7 H_2_O—0.0375, H_3_BO_3_—0.003, KI—0.015, MnSO_4_—0.75), YMG (glucose—20, malt extract—10, yeast extract—4), and SAB (glucose—40, peptone—10).

The fungal culture was also grown on a solid substrate: 100 g of pearl barley (PB), 60 mL of water in 500 mL Erlenmeyer flasks. All the media used in this study were sterilized by autoclaving at 121 °C for 20 min. Agar blocks (5 mm in diameter) cut from the edge of one-week *A. tenuissima* colonies obtained on PDA at 24 °C were used as seed inoculum (two blocks per flask). Incubation of the fungus was carried out at a constant temperature of 24 °C in the dark without agitation for three weeks for the liquid cultures, while the solid cultures were grown for two weeks and shaken once a day to improve aeration and prevent clumping. Three biological replicates were used for each culture medium.

### 2.2. Extraction of Secondary Metabolites

The mycelium was separated from the culture liquid by filtration through cheesecloth. Culture filtrates of *A. tenuissima* (300 mL each) were adjusted to pH 7 with 0.1 N NaOH and extracted with dichloromethane (DCM) (2 × 150 mL). Then remaining aqueous phases were adjusted to pH 3 with formic acid and repeatedly extracted with ethyl acetate (EtOAc) (2 × 150 mL). The dry solid culture was blended and extracted repeatedly with 300 mL of a mixture of acetone and water (50:50, *v*/*v*). After evaporation of the organic solvent, water phase was successively extracted with hexane and EtOAc twice for each solvent. The extracts were dried over anhydrous sodium sulfate and evaporated to dryness under vacuum at 40 °C.

### 2.3. Analysis of Extracts

Acetonitrile was used to dissolve the extracts samples to a concentration of 5 mg/mL. They were analyzed with an Acella high-performance liquid chromatography (HPLC) system with a diode-array detector and TSQ Quantum Access™ triple quadrupole mass spectrometer (Thermo Scientific, Waltham, MA, USA). The analysis was performed on a Zorbax SB-C18 column (Agilent Tech., Santa Clara, CA, USA, particle size 1.8 μm, 4.6 × 150 mm) with an eluent flow of 250 μL/min and a column temperature of 40 °C in a solvent system of acetonitrile–0.1%-formic acid in the gradient mode; acetonitrile concentration increased from 1 to 95% during the first 17 min, remained at 95% of acetonitrile for 3 min. The volume of the injected samples was 2 μL. The samples were analyzed in the positive mode in the scanning range of 100–1000 m/z using heated electrospray ionization. The retention time (t_R_), m/z values, and UV spectra of the metabolites were analyzed to compare the chromatographic profiles of the extracts.

The relationship between the composition of extracts (9 major compounds) obtained by different methods (5 media × 2 extraction solvents) and their biological activity (9 organisms) was assessed using principal component analysis (PCA) with data standardization prior to the analysis. Additional data on toxicity extracts to radish and *Paramecium caudatum* were taken from a published study [21].

### 2.4. Isolation of Tenuazonic Acid

The submerged fermentation of *A. tenuissima* MFP253011 was conducted in a 7 L bioreactor (Applikon Biotechnology, Delft, the Netherlands) containing 4.75 L of YMG medium for 7 days. A 250 mL volume of starter culture was grown in a 750 mL Erlenmeyer flask with the same medium that was inoculated with two agar blocks of the fungus colony on PDA (5 mm in diam) and incubated on a rotary shaker (at 180 rpm) at a temperature of 24 °C for 7 days. The conditions of submerged fermentation were as follows: incubation temperature 24 °C, aeration level 1 vvm, rotation speed 400 rpm, and initial pH 5.9 (not maintained at a constant level). Sunflower oil (1%, *v*/*v*) was added to the medium as an antifoam before the inoculation.

Extraction of metabolites from culture filtrate was performed as described in Section 2.2. The crude EtOAc extract (1272 mg) was separated on a glass column packed with Bio-Beads SX-8 resin (Bio-Rad, Hercules, CA, USA) using a mixture of DCM–EtOAc–formic acid (49.9:49.9:0.2, *v*/*v*) as the eluent with a flow rate of 5 mL/min. The resulting TeA-containing fraction II (890 mg) was applied to a Chromabond C18ec cartridge (10 g, Macherey-Nagel, Düren, Germany), and the column was eluted sequentially with 40 mL of MeOH–0.1% formic acid mixture (25:75, 50:50, 75:25, 100:0, *v*/*v*), yielding five subfractions (A–E). Subfraction B (775 mg) was further purified by preparative HPLC to afford TeA (475 mg) as yellow oil (XBridge Prep C18 5 μm, column size 19 × 250 mm, elution with 30% acetonitrile in 0.1% formic acid, flow rate 24 mL/min, detection 272 nm, t_R_ 11.2 min). The following chromatographic system was used for preparative HPLC: Quaternary Gradient Module 2545, UV/Visible Detector 2489, and Fraction Collector III (Waters, Milford, MA, USA). ^1^H and ^13^C NMR spectra were recorded at 400 and 100 MHz, respectively, in CDCl_3_ as a solvent on a Bruker AVANCE III 400 MHz spectrometer (Bruker, Karlshrue, Germany). The solvent residual signal (δ 7.26 ppm) for ^1^H NMR spectra and the carbon signal of CDCl_3_ (δ 77.16 ppm) for ^13^C NMR spectra were used as references. ESI-MS spectra were recorded with a TSQ Quantum Access spectrometer (Thermo Scientific, Waltham, MA, USA) after HPLC as described in Section 2.3. The spectral and spectroscopy data are given in the Supplementary Information. TeA was identified using literature data [22].

Tenuazonic acid: ESI-MS m/z 198.02 [M + H]^+^; ^1^H NMR, predominant form: 6.77 br.s (NH), 3.8 d (3.5 Hz), 2.47 s, 1.98 m, 1.40 m, 1.25 m, 1.03 d (7.0 Hz), 0.97 t (7.35 Hz); minor form: 6.65 br.s (NH), 3.97 d (3.8 Hz), 2.52 s, 1.98 m, 1.41 m, 1.31 m, 1.03d (7.0 Hz), 0.97 t (7.35 Hz); ^13^C NMR, predominant form: 195.4, 184.4, 176.3, 102.5, 67.1, 37.3, 23.7, 19.7, 15.8, 11.7; minor form: 201.1, 189.0, 169.8, 106.0, 63.8, 37.1, 24.0, 20.6, 15.4, 11.7.

### 2.5. Bioassays

#### 2.5.1. Entomotoxic Activity of Extracts

The toxicity of *A. tenuissima* extracts was evaluated against the spring green aphid (*Schizaphis graminum*) and greater wax moth (*Galleria mellonella*) supplied by the Laboratory of Biological Control at the All-Russian Institute of Plant Protection. *S. graminum* was reared on wheat seedlings at 25 ± 1 °C and 65 ± 5% relative humidity, while *G. mellonella* was kept in the dark at 30 °C in plastic boxes with perforated lids filled with artificial feed (90 g corn grits, 40 g wheat flour, 50 g milk powder, 10 g yeast, 50 g glycerol, 50 g beeswax, 50 mL water). Prior to the bioassays, the extracts samples were dissolved in ethanol, and then the solutions were adjusted with water to an extract concentration of 5 mg/mL, while the final concentration of ethanol was 5% (*v*/*v*).

The contact intestinal aphicidal bioassay was described in detail earlier [23]. Briefly, filter paper disks (4 cm in diameter) placed on the bottom of a Petri dish were moistened with 0.5% extracts (250 μL per disk or 1 mg/dm^2^). Segments of wheat leaves (2 cm length) were dipped in the same test solutions and were placed on the filter paper. In the control treatment, 5% aqueous ethanol (*v*/*v*) was used. Twenty aphids were transferred to each Petri dish. The mortality percentage of tested insects was recorded 24 h post-treatment under the above-mentioned conditions. Four replicates were made for each extract.

For the evaluation of the acute contact larvicidal activity of the extracts, the injection method was used as described previously [24] with slight modifications. Briefly, 10 μL of 0.5% extracts were injected into the hemocoel via the third segment of IV‒V instar *G. mellonella* larvae (190–230 mg each) with a Hamilton syringe. As a control treatment, 5% aqueous ethanol (*v*/*v*) was used. After the extract injection, the larvae were placed in a 90 mm sterile Petri dish supplemented with 2 g of artificial feed and were incubated in the dark for 10 days to record mortality daily. Twenty *G. mellonella* larvae were tested per treatment, and the experiment was repeated twice.

#### 2.5.2. Phytotoxic Activity of Extracts

The phytotoxic activity of the extracts was assayed using the previously described method [25]. Leaf discs (1 cm in diameter) of perennial sowthistle (*Sonchus arvensis*) and leaf segments (2 cm length) of wheat (*Triticum aestivum*) were placed in a wet chamber and accurately punctured by a sharp needle. A 10 μL droplet of 0.5% extract prepared in 5% ethanol was placed above the puncture. In the control treatment, 5% aqueous ethanol was used. The phytotoxic activity was determined as the diameter or length of necrotic lesions (for sowthistle and wheat, respectively) after 48 h of exposure at 24 °C and a 12 h photoperiod. Six leaf disks/segments were used for each treatment.

#### 2.5.3. Cytotoxic Activity of Extracts

The cytotoxicity of *A. tenuissima* extracts was assayed on the Sf9 cell line (ECACC 89070101) originating from the ovarian tissue of the fall armyworm (*Spodoptera frugiperda*) maintained in the Laboratory of Molecular Plant Protection at the All-Russian Institute of Plant Protection. Samples (10 μL) of assayed extracts dissolved in dimethyl sulfoxide (DMSO) to a concentration of 10 mg/mL were added to 890 μL of fresh SF900II culture medium (Thermo Fisher Scientific, Waltham, MA, USA) and 100 μL of a suspension of actively growing cells (1 × 10^5^ cells/mL, viability ≥ 90%) in wells of a 48-well plate. The final concentration of the solvent was 1%. A 1% DMSO solution was used as a control treatment. The cells were incubated for 24 h at 27 °C and were then stained with 0.4% aqueous solution of trypan blue to count the percentage of dead (stained) cells in relation to the total number (at least 50) in several fields of view. Three replicates were used for each extract.

#### 2.5.4. Antimicrobial Activity of Extracts

The antimicrobial activity of *A. tenuissima* extracts was tested against *Bacillus subtilis* NCTC 104000 and *Candida* *albicans* NCPF 3179 using the paper disk agar diffusion assay [26]. The microorganisms were grown on PDA. The samples of assayed extracts were dissolved in acetone and applied to the 6 mm paper discs (Macherey-Nagel, Düren, Germany) at a concentration of 500 µg/disk. The treated microbial cultures were incubated at 30 °C for 24 h before the activity was determined as the radius of the growth inhibition zone in mm. Three replicates were used for each extract.

#### 2.5.5. Insect Models and TeA

*G. mellonella* larvae and *S. graminum* females were prepared as described in Section 2.5.1. The *Zophobas morio* laboratory population was reared in trays with wheat bran and fruit peelings. The colony of house cricket (*Acheta domesticus*) was reared in plastic boxes filled with carton pieces and fruit peelings. The population of the two-spotted spider mite (*Tetranychus urticae*) was grown on common bean (*Phaseolus vulgaris*) seedlings. All arthropods were obtained from the abovementioned laboratory of biological control.

The samples of TeA were dissolved in ethanol for bioassays as described above. The analytical standard (Sigma-Aldrich, St. Louis, MO, USA) of the entomotoxic fungal metabolite beauvericin was used as a positive control in some bioassays in the same way as TeA.

#### 2.5.6. Acute Contact Toxicity of TeA

An injection test was performed using the IV–V instar larvae of *G. mellonella* (190–230 mg each) and *Z. morio* (135–150 mg each); the procedure was similar to that described in Section 2.5.1. The concentrations of TeA solutions for injection were 0.5, 1, 2, and 5 mg/mL. After injection, test insects were placed in sterile Petri dishes supplemented with 2 g of feed (the artificial feed for *G. mellonella* and a piece of zucchini for *Z. morio*) and incubated in darkness at 30 °C for ten days. The mortality and weight of treated larvae were recorded daily. Five larvae were tested as a group within the experiments, and four replicates were performed.

#### 2.5.7. Oral Toxicity of TeA

*G. mellonella* larvae and *A. domesticus* adults were subjected to a diet containing different concentrations of TeA. A bioassay was conducted by dissolving TeA in acetone and adding it to food samples (artificial feed for *G. mellonella* and a piece of zucchini for *A. domesticus*) at concentrations of 0.25, 1.0, and 2.5 mg/g. The control was treated with the same volume of acetone. The solvent was evaporated from treated feed samples at room temperature for 45 min. Beginning on the second day of the experiment, the feed samples were replaced with fresh ones every two days. Ten larvae of *G. mellonella* were tested as a group within the experiments, and two replicates were performed. Ten *A. domesticus* adults were tested per replicate, and four replicates were performed. The insects were incubated for 10 days in darkness. The mortality and weight of the insects were recorded daily.

#### 2.5.8. Contact Intestinal Toxicity of TeA

Aphicidal activity of TeA was tested against the wheat aphid as described above (Section 2.5.1) at concentrations of 0.5, 1.0, and 2.0 mg/mL (100, 200, and 400 µg/dm^2^). The acaricidal effects of TeA were tested against the mite *T. urticae* at the same concentrations, similar to a published technique [27]. Common bean leaf discs (diameter of 4 cm) were dipped for 2 s in the toxin solutions. After liquid evaporation, the leaf discs were placed in a glass Petri dish (diameter of 9 cm) on filter paper moistened with water. In the control treatment, leaf discs were dipped in 5% ethanol as described above. Twenty *T. urticae* females were introduced to each leaf replicate disc, and five replicates were performed.

#### 2.5.9. Cytotoxic Activity of TeA

Sf9 cells were used for TeA cytotoxicity assessment at a concentration range of 1–100 μg/mL, as described in Section 2.5.3.

### 2.6. Statistical Analysis

Statistical analysis was performed using Statistica 10 (StatSoft, Tusla, OK, USA) and SigmaPlot 14 (Systat Software, San Jose, CA, USA) software. Data normality was determined by the Shapiro–Wilk W test. The values expressed as percentages (%) were transformed (l g or square root) before the tests. Normally distributed data were analyzed using one- or two-way analysis of variance (ANOVA) depending on the experimental setup. Pairwise comparison was performed by Tukey’s HSD test at a significance level of *p* = 0.05. In the absence of a normal distribution, the data were analyzed by methods of nonparametric statistics. The significance of the factor effects was determined using a Kruskal–Wallis H-test (one-way ANOVA on ranks), and significant differences between the median values were determined by Dunn’s post hoc test. The survival rate of arthropods under the influence of TeA was determined by log-rank test with Holm–Sidak adjustment. IC_50_ values representing TeA concentration required to cause a 50% reduction in Sf9 cells viability were determined by the SigmaPlot curvilinear regression procedure.

## 3. Results

### 3.1. The Yield of A. tenuissima Extracts

**Figure 1:**
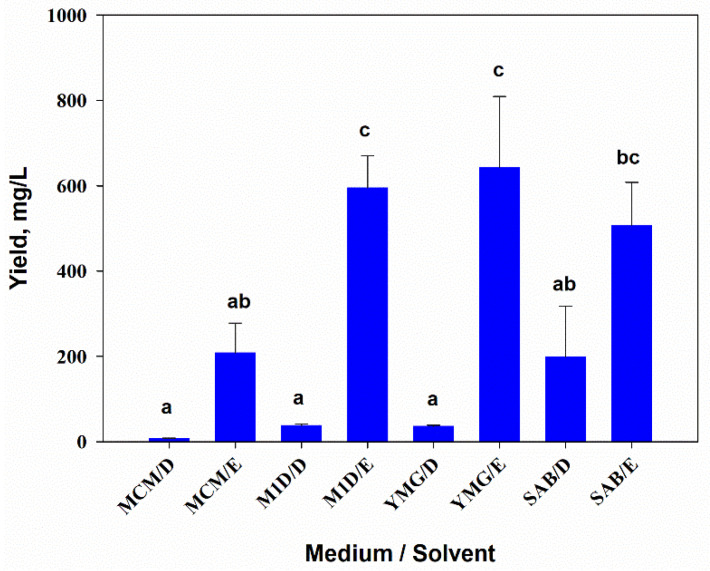
Yield of extractive matter from the filtrate of *Alternaria tenuissima* MFP253011 cultures on various liquid media obtained by successive extractions with dichloromethane (D) and ethyl acetate (E). Means ± standard deviation (*n* = 3) marked with the same letter did not differ significantly at the level of *p* = 0.05 according to Tukey’s HSD test.

The results of two-way ANOVA showed that the composition of the nutrient medium (F_3,16_ = 6.1, *p* = 0.01) and the extraction solvent (F_1,16_ = 79.8, *p* = 0.001) significantly affected the yield of extractive matter (YEM) from the culture filtrate of *A. tenuissima* MFP253011. The interaction of both factors was also significant (F_3,16_ = 4.4, *p* = 0.02). The YEM from the fungal culture filtrate cultivated on MCM was almost three times lower than those grown on other liquid media. When sequentially extracted with DCM and EtOAc, the YEM was on average seven times higher in EtOAc extracts than in DCM extracts. The maximum yield of DCM extracts (about 200 mg/L) was obtained by growing the fungus on SAB medium. YEM of EtOAc extracts was maximal when YMG medium (more than 640 mg/L) was used (Figure 1).

When the fungus was cultured on pearl barley, the yield of the hexane extracts was 408.3 ± 40 mg/kg, while EtOAc extracts yielded 1740.3 ± 49.8 mg/kg.

### 3.2. The Spectrum of Biological Activity of A. tenuissima Extracts

#### 3.2.1. Entomotoxic Activity

**Figure 2:**
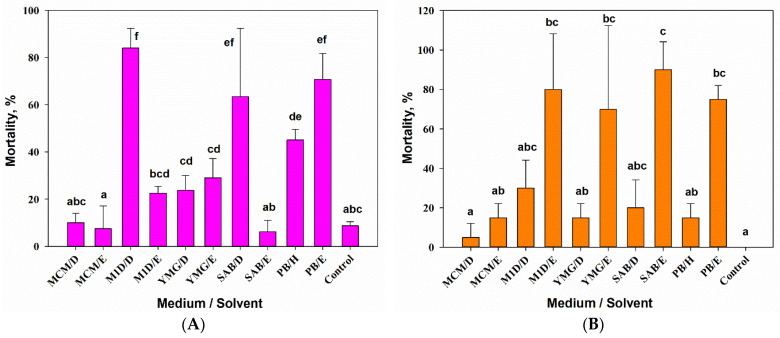
Entomotoxic activity of extracts from *Alternaria tenuissima* MFP253011 cultures on various liquid and solid nutrient substrates on *Schizaphis graminum* at a concentration of 5 mg/mL 24 h post treatment (**A**) and on *Galleria mellonella* at a concentration of 50 µg/larva 7 days post treatment (**B**). Means ± std. deviation marked with the same letter did not differ significantly at the level of *p* = 0.05 according to Tukey’s HSD test. D—dichloromethane, E—ethyl acetate, H—hexane.

The results of one-way ANOVA showed that the composition of the substrate and the extraction solvent as a combined factor had a significant (F_10,33_ = 25.37, *p* = 0.01) effect on the aphicidal activity of *A. tenuissima* MFP253011 extracts. The maximum activity against *S. graminum* (>80% mortality 24 h post-treatment) was shown by the DCM extract from the filtrate of the *A. tenuissima* culture on the M1D medium. The common wheat aphid was nonsignificantly (at *p* = 0.05) less sensitive to the DCM extract from the filtrate of the *A. tenuissima* culture on SAB (~70% mortality) and the EtOAc extract from the solid culture of the fungus (>60% mortality). Hexane extract from the solid culture of *A. tenuissima* also had aphicidal activity (~50% mortality) that was significantly (at *p* = 0.05) different from the control values. The sensitivity of the common wheat aphid to other extracts was low (≤25% mortality) and did not differ from the control at *p* = 0.05 (Figure 2A).

The composition of the nutrient medium for growing *A. tenuissima* and the extraction method had a significant effect (one-way ANOVA, F_9,10_ = 7.35, *p* = 0.01) on the toxicity of extracts to *G. mellonella* larvae. Most of the dead larvae were melanized 3–4 days post treatment. No larvae died in the untreated control by the 7th day of observation. A high level of larvicidal activity (≥75% mortality 7 days post-treatment), which was significantly different from the control at *p* = 0.05, was demonstrated by the EtOAc extracts from the cultures produced on various liquid (M1D, YMG, SAB) and solid substrates. Among the DCM extracts, the maximum toxicity (only 30% larval mortality), which differed from the control at *p* = 0.05, demonstrated an extract from culture liquid on M1D. In the remaining extracts, the larvicidal activity was noticeably low, the mortality rate was below 30%. No larvae were affected in the control (Figure 2B).

#### 3.2.2. Phytotoxic Activity

All the evaluated extracts of *A. tenuissima* were toxic to leaf segments of both wheat and perennial sowthistle. At the same time, the medium composition combined with the extraction solvent had a significant (*p* = 0.001) effect on phytotoxic activity of the fungal extracts for wheat (H_9,50_ = 49.1) and sowthistle (H_9,50_ = 45.9) segments according to Kruskal–Wallis one-way ANOVA on ranks. The EtOAc extracts from the filtrate of *A. tenuissima* cultures grown on M1D, YMG, and SAB media showed relatively high activity on the leaf segments of both plants. No phytotoxicity symptoms were found in the control treatments.

**Figure 3:**
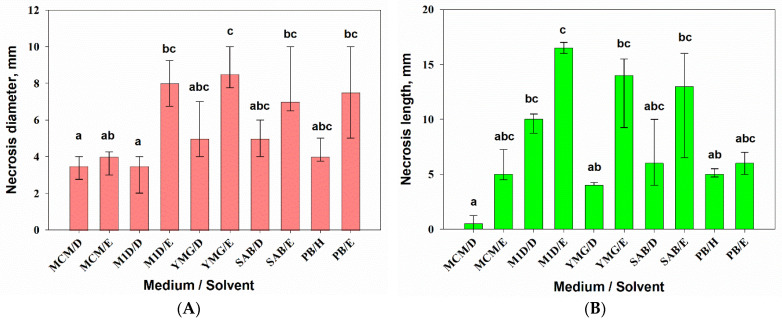
Phytotoxic activity of 0.5% extracts from *Alternaria tenuissima* MFP253011 cultures grown on various liquid and solid media 48 h post-treatment assayed on leaf discs of *Sonchus arvensis* (**A**) and leaf segments of *Triticum aestivum* (**B**). Median values with 25–75% quartiles marked with the same letter did not differ significantly at the level of *p* = 0.05 according to Dunn’s test. D—dichloromethane, E—ethyl acetate, H—hexane.

The leaf discs of sowthistle were highly sensitive (necrosis diameter 7–9 mm) to EtOAc extracts from *A. tenuissima* cultures grown on most of the used substrates, with the exception of MCM. DCM extracts from culture liquid and hexane extract from solid culture were generally less toxic; the diameter of necrosis was in the range of 3–5 mm (Figure 3A). The EtOAc extract from the filtrate of culture on YMG was significantly more toxic compared with the DCM extract from the culture liquid produced on M1D medium (*p* = 0.002) and the DCM (*p* = 0.03) and EtOAc extracts from cultures on MCM (*p* = 0.021).

There were significant differences in sensitivity of wheat leaf segments to highly toxic EtOAc extract from the M1D liquid culture of *A. tenuissima* and three non-polar extracts: the DCM extracts from MCM (*p* =0.001) and YMG (*p* = 0.01) liquid cultures as well as the hexane extract from PB solid culture (*p* = 0.03) (Figure 3B).

#### 3.2.3. Cytotoxic Activity

**Figure 4:**
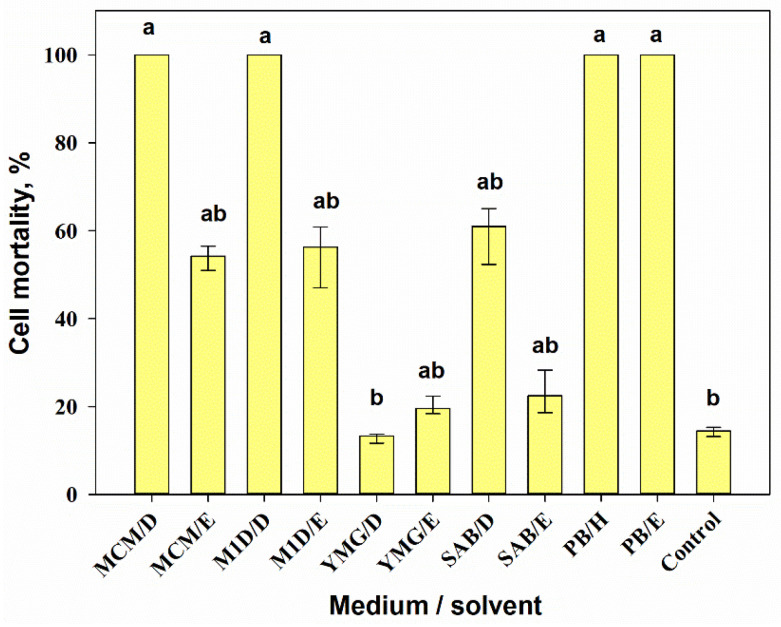
Cytotoxic activity of 0.01% extracts from *Alternaria tenuissima* MFP253011 cultures grown on various liquid and solid media 24 h post-treatment assayed on Sf9 cells. Median values with 25–75% quartiles marked with the same letter did not differ significantly at the level of *p* = 0.05 according to Dunn’s test. D—dichloromethane, E—ethyl acetate, H—hexane.

Combining the medium composition and the extraction method had a significant effect (H_10,33_ = 41.9, *p* = 0.001) on the cytotoxicity of 0.01% extracts from *A. tenuissima* cultures according to Kruskal–Wallis one-way ANOVA on ranks. DCM extracts from the filtrate obtained from MCM and M1D liquid cultures, as well as hexane and EtOAc extracts from the solid (PB) culture of *A. tenuissima,* caused 100% mortality of Sf9 cells. The cytotoxicity of the DCM extract from the YMG liquid culture was significantly lower (*p* = 0.014) than the toxicity of the mentioned extracts. The EtOAc extracts from the filtrate obtained from MCM and M1D liquid cultures and the DCM extract from the filtrate of fungal liquid cultures grown on SAB were moderately toxic (50–60% cell mortality). EtOAc extracts from the filtrate of fungal liquid cultures grown on semi-synthetic YMG and SAB media were weakly toxic (up to 20% cell mortality) (Figure 4).

#### 3.2.4. Antimicrobial Activity

**Figure 5:**
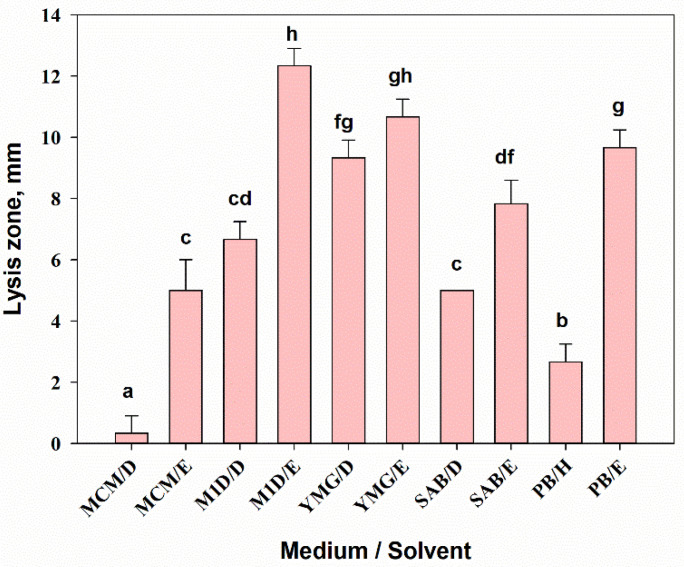
Antibacterial activity of extracts from *Alternaria tenuissima* MFP253011 cultures grown on various liquid and solid media 24 h post-treatment against *Bacillus subtilis*. Means ± std. deviation marked with the same letter did not differ significantly at the level of *p* = 0.05 according to Tukey’s HSD test. D—dichloromethane, E—ethyl acetate, H—hexane.

The medium composition and the extraction method had significant effects (one-way ANOVA, F_9,20_ = 107.6, *p* = 0.001) on the antibacterial activity of *A. tenuissima* extracts against the gram-positive bacterium *Bacillus subtilis*. The highest inhibitory activity (10–12 mm lysis zone) was shown by EtOAc extracts (500 µg/disc) from *A. tenuissima* cultures grown on M1D and YMG media as well as on the solid substrate. The bacterium was also moderately sensitive to DCM extracts from the culture fluid of the fungus on M1D and YMG media (the radius of the lysis zone was 7–9 mm) (Figure 5). These two extracts considerably suppressed the growth of *C. albicans* (9–11 mm lysis zone), while the activity of others was insignificant (data not shown).

### 3.3. Analysis of Extracts

In the extracts from the culture liquid of *A. tenuissima* MFP253011, three known mycotoxins (tentoxin, dihydrotentoxin, and tenuazonic acid) were detected by characteristic MS- and UV-spectra. Another group of substances presumably belonged to the meroterpenoid group, such as ACTG toxins and tricycloalternarenes (chromatography peaks with m/z 321, 345, 349, 363, and 377 and a typical 266–272 nm single UV-absorption band). Their exact identification in the extracts is difficult because of the presence of isomers [28,29,30,31,32].

**Figure 6:**
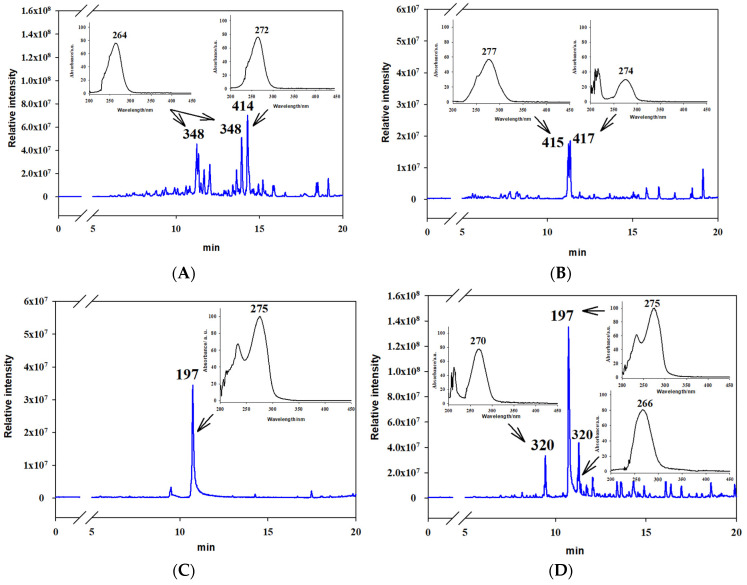
Representative HPLC/MS chromatograms of extracts showing entomotoxic activity from *Alternaria tenuissima* MFP253011 cultures on various media obtained with dichloromethane (**A**) M1D medium, (**B**) Sabouraud medium and ethyl acetate, (**C**) YMG medium, and (**D**) pearl barley.

Three tricycloalternarene (TCA) compounds with an mw of 348 Da (presumably isomers of TCA 1) and 362 Da (presumably TCA 11 a/b) as well as a polypeptide (presumably iso-tentoxin with an mw of 414 Da) were identified as major metabolites in the DCM extract from fungal culture on M1D liquid medium (Figure 6A), which showed aphicidal and other types of activity (Figure 2A).

When *A. tenuissima* was cultured on semi-synthetic YMG and SAB media, tentoxin and dihydrotentoxin prevailed in DCM extracts from the culture liquid. The latter extract was more complex and contained two TCAs (presumably TCA 4 a/b and TCA 5 a/b) with an mw of 344 Da (Figure 6B) and showed aphicidal activity (Figure 2A).

In the EtOAc extracts of *A. tenuissima*, which were highly toxic to *G. mellonella* larvae (Figure 2B), the major metabolite was tenuazonic acid. Its relative content was high in the extracts from the filtrate of cultures on M1D, YMG, and SAB media, while the EtOAc extract from the solid culture had a relatively lower concentration of the toxin because of the presence of other compounds, such as two substances with mw 320 Da (presumably, TCA A isomers) (Figure 6C,D).

Data on the biological activity of 10 extracts on nine test organisms and the relative content of nine major metabolites in them were evaluated with PCA. Three principal components (PC) were revealed, which explained 40.1, 18.5, and 12.6% of the data variance, respectively; the effect of the remaining seven components to explain the data was not significant.

**Figure 7:**
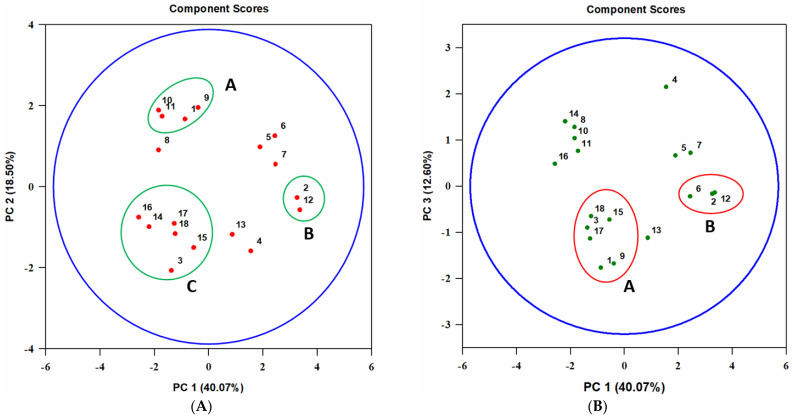
Principal component (PC) analysis (**A**)–PC1 vs. PC2, (**B**)–PC1 vs. PC3 on the relationship between the sensitivity of various organisms to different *A. tenuissima* MFP253011 extracts and the contents of major metabolites in them. Test organisms: **1**—*Schizaphis graminum*, **2**—*Galleria mellonella*, **3**—Sf9 cell line, **4**—*Raphanus sativus*, **5**—*Triticum aestivum*, **6**—*Sonchus arvensis*, **7**—*Bacillus subtilis*, **8**—*Candida albicans*, **9**—*Paramecium caudatum*. Analyzed compounds (molecular weight, Da): **10**—tentoxin (414), **11**—dihydrotentoxin (416), **12**—tenuazonic acid (197), **13**—unidentified (320), **14**—unidentified (328), **15**—unidentified (338), **16**—unidentified (344), **17**—unidentified (348), **18**—unidentified (362).

Analyzing PC1 and PC2, it was found that the sensitivity of the common wheat aphid *S. graminum* and the infusoria *P. caudatum* to some extracts may be associated with the content of tentoxin and dihydrotentoxin (see cluster A in Figure 7A). In the PC1 and PC3 coordinates, the aphicidal and cytotoxic activity of the extracts seemed to be associated with the presence of the metabolites with molecular weights of 338, 348, and 362 Da (see cluster A on Figure 7B). The sensitivity of *G. mellonella* larvae to the extracts was closely correlated with the content of tenuazonic acid (TeA) (see cluster B in Figure 7A,B).

The sensitivity of Sf9 cells appeared to be correlated with TCA content in the extracts (cluster C in Figure 7A, cluster A in Figure 7B).

Considering the relatively high yield of EtOAc extracts (Figure 1), TeA was found to be the main easily available exo-metabolite in the liquid cultures of *A. tenuissima* MFP253011 grown on M1D and YMG media. Because these extracts demonstrated relatively high toxicity to *G. mellonella* larvae, TeA was purified from them to confirm the entomotoxic activity.

### 3.4. Entomotoxic Activity of Tenuazonic Acid

#### 3.4.1. Acute Contact Larvicidal Toxicity

**Figure 8:**
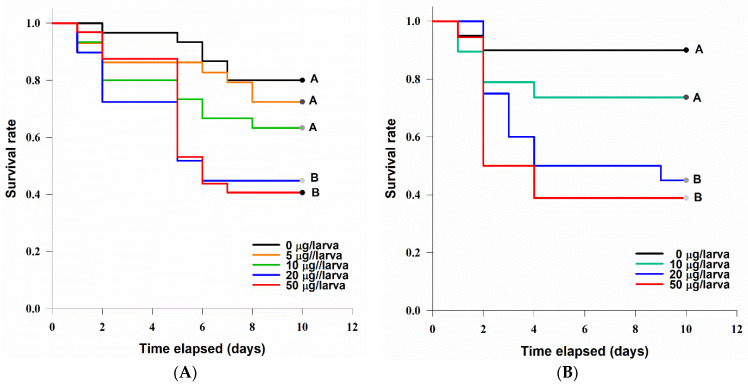
Survival curves of *Galleria mellonella* (**A**) and *Zophobas morio* (**B**) larvae after injection with various concentrations of tenuazonic acid. The curves marked with one letter did not differ significantly at *p* = 0.05 by log-rank test with Holm–Sidak adjustment.

*G. mellonella* larvae were sensitive to the injection of TeA in the tested concentrations. The dead caterpillars became melanized. A statistically significant difference between the survival curves was revealed (log-rank test: χ^2^ = 15.5, df = 4, *p* = 0.004). At concentrations of 20 µg/larva (LT_50_ = 6 ± 1.3 days) and 50 µg/larva (LT_50_ = 6 ± 0.8 days), TeA significantly (at *p* = 0.05) decreased the survival rate of *G. mellonella* larvae compared with the control (Figure 8A).

*Zophobas morio* larvae were also sensitive to the injection of various concentrations of TeA (Figure 8B). There was a statistically significant difference between the survival curves (log-rank test: χ^2^ = 12.7, df = 3, *p* = 0.005). At concentrations of 20 µg/larva (LT_50_ = 4 ± 4.5 days) and 50 µg/larva (LT_50_ = 2 ± 0.6 days), TeA significantly (at *p* = 0.05) decreased the survival rate of *Z. morio* larvae compared with the control.

#### 3.4.2. Oral Toxicity

**Figure 9:**
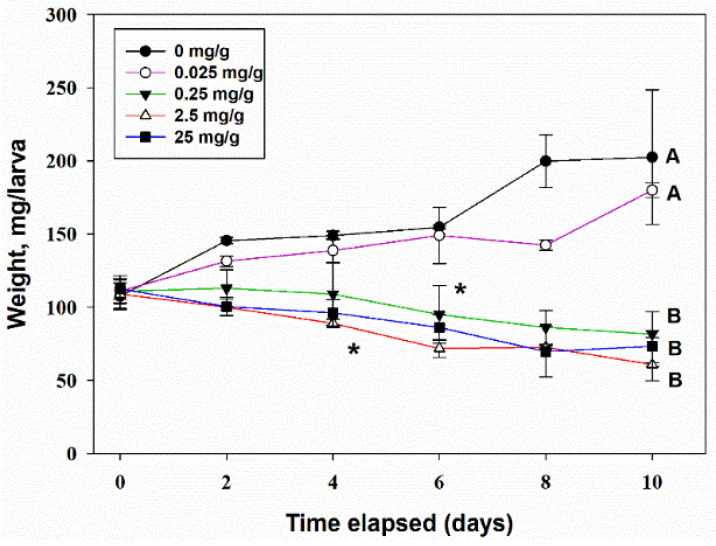
The effect of the concentration of tenuazonic acid in artificial feed on the growth of the biomass of *Galleria mellonella* larvae. Means ± std. deviation marked with the same letter did not differ significantly at the level of *p* = 0.05 according to Tukey’s HSD test. *—significant difference from control at *p* = 0.05 by Dunnett’s test.

Two-way ANOVA of the lg-transformed data showed that TeA concentration in the artificial feed strongly (F_4,30_ = 70.1, *p* < 0.001) affected the biomass of *G. mellonella* larvae, while the time factor was insignificant (F_5,30_ = 1.4, *p* = 0.25). The interaction of the factors “time” and “TeA concentration” was significant (F_20,30_ = 6.4, *p* < 0.001), indicating the existence of different trends. A significant (at *p* = 0.05) decrease of larvae biomass accumulation compared with the control was noted at a TeA concentration of 2.5 mg/g of the feed, starting from the 4th day post-treatment; by the 10th day of observations, it became approximately two times lower than in the control. At a concentration of 0.25 mg/g of feed, the inhibitory effect of TeA was pronounced on the 6th day of the experiment (Figure 9). Visually, *G. mellonella* larvae refused to eat the feed containing TeA. This probably explains why their mortality did not exceed the level of 20% by the 10th day of observations at a TeA concentration of 25 mg/g of feed.

**Figure 10:**
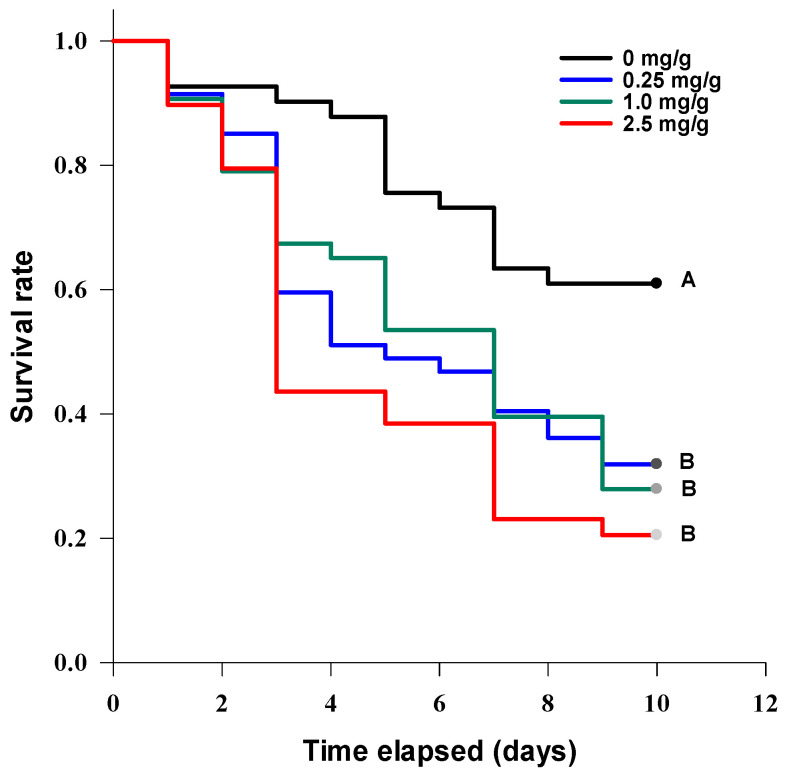
The survival curves of *Acheta domesticus* adults at different concentrations of tenuazonic acid added to the feed. The curves marked with one letter did not differ significantly at *p* = 0.05 by log-rank test with Holm–Sidak adjustment.

After 2 days of incubation, *Acheta domesticus* adults completely consumed the toxin-spiked and control feed, and in all the treatments it was replaced with fresh (without the addition of TeA) feed. A statistically significant difference (log-rank test: χ^2^ = 16.4, df 3, *p* = 0.001) between the survival curves of the crickets fed with different TeA contents was revealed (Figure 10). The cricket mortality rate was significantly (at *p* = 0.05) higher than that of the control at all TeA concentrations used, but the differences between the treatment options were insignificant. The median survival of the cricket was 3 ± 0.2, 5 ± 1.5, and 7 ± 0.9 days at toxin concentrations of 2.5, 1, and 0.25 mg/g of feed, respectively.

#### 3.4.3. Contact Intestinal Activity

**Figure 11:**
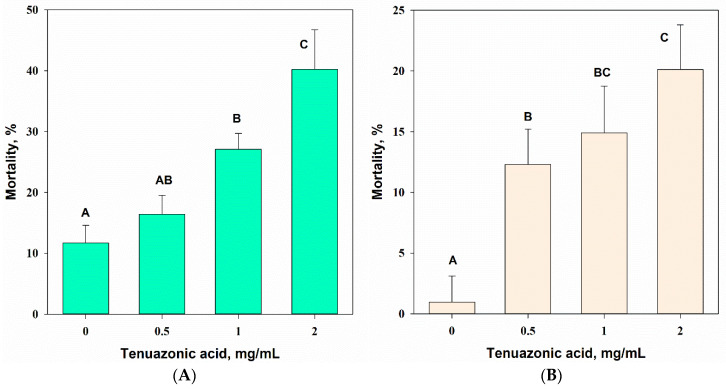
Toxicity of various concentrations of tenuazonic acid against *Schizaphis graminum* (**A**) and *Tetranichus urticae* (**B**) 24 h post treatment. Means ± std. deviation marked with the same letter did not differ significantly at the level of *p* = 0.05 according to Tukey’s HSD test.

The concentration of TeA had a significant (one-way ANOVA, *p* = 0.001) effect on the mortality of the common wheat aphid (F_3,8_ = 28.4) and female of spider mites (F_3,16_ = 31.4) 24 h after treatment. Significant (at *p* = 0.05) aphicidal activity of TeA was noted at a toxin concentration of 1.0 mg/mL (27% mortality), and acaricidal action was revealed at a concentration of 0.5 mg/mL (12% mortality). At a maximal concentration of 2 mg/mL, the mortality reached 43% and 19% for the aphids and mites, respectively (Figure 11A,B). Beauvericin caused 100% mortality of the tested arthropods at a concentration of 0.5 mg/mL.

No symptoms of phytotoxicity were manifested on wheat leaf segments soaked in TeA solution, whereas small necrotic lesions (up to 10% of total leaf disc area) were noted on bean leaves at a TeA concentration of 2 mg/mL 24 h after treatment.

#### 3.4.4. Cytotoxic Activity

**Figure 12:**
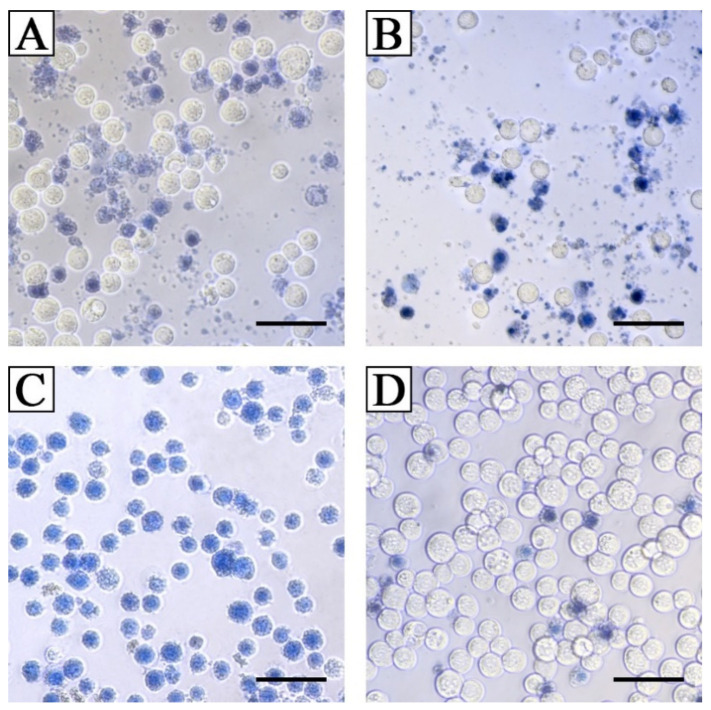
Morphology of Sf9 cells after 24 h of exposure with 15 μg/mL (**A**) and 25 μg/mL (**B**) of tenuazonic acid, and 25 μg/mL of beauvericin (**C**); (**D**) control (1% DMSO). Bar indicates 50 μm.

TeA was approximately five times less toxic with IC_50_ 25 µg/mL (126 µM) than beauvericin with IC_50_ 4 µg/mL (5 µM) against Sf9 *Spodoptera frugiperda* cells. Unlike beauvericin, at all the studied concentrations, TeA caused rapid degradation of Sf9 cells. It should be noted that the exact calculation of the IC_50_ for TeA was difficult due to the fact that the dead cells were rapidly destroyed (Figure 12).

## 4. Discussion

### 4.1. Detection of Entomotoxic Metabolites in A. tenuissima Extracts

Metabolites of small-spored *Alternaria* species have been characterized as mycotoxins (tenuazonic acid, alternariol and its methyl ester, altertoxins) and as substances with phytotoxic (e.g., altenuene, tenuazonic acid, tentoxin, and a number of host-specific toxins), antimicrobial (e.g., altersetin), cytotoxic activity (bi- and tricycloalternarenes), and other interesting properties (enzyme inhibitors, antioxidants, etc.). Many of them possess several types of biological activity [33,34,35]. In the present and earlier studies [21], the extracts from *A. tenuissima* MFP253011 cultures obtained by various methods (using different media and solvents) were found to display entomotoxic activity along with phytotoxic, antimicrobial, and cytotoxic properties (Figure 2, Figure 3, Figure 4 and Figure 5). A number of known (tenuazonic acid, tentoxin, and dihydrotentoxin) and unidentified compounds belonging to the tricycloalternarene group were detected, but the composition of extracts varied depending on the production method (Figure 6). This allowed us to evaluate the relationship of the entomotoxic activity of *A. tenuissima* extracts with other bioactivity types together with the formation of nine major metabolites (Figure 7). A similar approach was successfully implemented to identify the determinants of the pathogenicity of *Ilyonectria mors-panacis* affecting ginseng [36] and the biological activity of the aquatic micromycete *Aspergillus awamori* [37].

Analysis of the composition of extracts and their activity by PCA revealed that the aphicidal activity of extracts was associated with the presence of tentoxin and dihydrotentoxin as well as some tricycloalternarenes (TCA), for example, TCA 1 a/b (mw 348 Da), TCA 11 a/b (mw 362 Da), and others (Figure 7). However, the entomotoxic activity of the compounds is unknown, while their cytotoxic activity has not been sufficiently studied. For instance, weak cytotoxicity of tentoxin against HepG2 [38] and HeLa cell lines [39] as well as some TCAs for various tumor cell lines [40,41] have been reported. A relationship between the production of TCAs and the cytotoxic activity of *A. tenuissima* MFP253011 extracts against Sf9 cells is more likely (Figure 7). The phytotoxicity of tentoxin [42] and TCA 1 a/b [43] to wheat, the host plant of *S. graminum*, may be responsible for the indirect effect of these substances on the aphid. In general, the spectrum of biological activity of tentoxin, dihydrotentoxin, and TCAs is still poorly understood [44], and an evaluation of their anti-insectan properties may be interesting. However, the yield of extracts containing these metabolites was relatively low (up to 200 mg/L) (Figure 1). In this regard, this work may be planned for the future with optimized media conditions for their production.

PCA indicated that tenuazonic acid (TeA) was responsible for the larvicidal toxicity of some EtOAc extracts against *G. mellonella* (Figure 7), a model insect for studying the effects of toxins, immunosuppressants, and antibiotics as well as the relationship between pathogens and insects [45]. According to the literature, TeA was entomotoxic to the first instar larvae of the green bottle fly *Lucilia sericata* (LD_50_ 120 µg/mL) but was not active against other tested arthropods from various orders, namely *Drosophila melanogaster* (Diptera), *Sitophilus granarius* (Coleoptera), *Aphis fabae* (Hemiptera), and *Tetranychus urticae* (Trombidiformes) [11]. There are no data on the sensitivity of *G. mellonella* to this toxin. In order to study the entomotoxic properties of TeA, it was isolated from the culture of *A. tenuissima* MFP253011.

In liquid cultures of various strains of small-spored *Alternaria* spp., TeA content was detected with a yield of approximately 20–40 mg/L. Commonly, the producer strains were grown on both mineral (Richard’s and Czapek) and semi-synthetic (potato-dextrose broth, YES, and Richard’s + 0.5% peptone) liquid media. The last medium allowed maximum toxin yields of up to 100–300 mg/L. When the fungi were grown on solid substrates, the yield of TeA reaches ~200 mg/kg [46,47,48,49,50]. TeA was found to be a major toxin (up to 79% of the total content) in extracts of some *A. tenuissima* strains [51]. In our study, a relatively high yield of TeA was obtained (105 mg/L, ~17% of the extract) by submerged fermentation of *A. tenuissima* MFP253011 in a bioreactor on YMG medium, which stimulated the fungus to produce the relatively high yield of extractive matter (Figure 1), and by using the standard procedures for toxin isolation from the culture liquid. This may indicate that TeA is an easily available metabolite of *A. tenuissima*, which can be used for various biological assays requiring large amounts of the substances like entomotoxicity tests.

### 4.2. Entomotoxicity of Tenuazonic Acid

The injection method using *G. mellonella* larvae was successfully tested for the toxicological characterization of various chemicals [24]. For example, okadaic acid from shellfish at concentrations of ≥75 ng/larva led to a significant decrease in the survival rate of *G. mellonella* (>65% morality) 24 h after injection with an LD_50_ of ~239 µg/kg, comparable to its toxic dosage for rats [52]. This method has been used to assess the acute toxicity of metabolites of entomopathogenic fungi. In particular, a purified mixture of efrapeptins from *Tolypocladium* spp. was toxic to *G. mellonella* with an LD_50_ of 30 ng/larva [53]. At a dosage of 8.6 µg/larva, beauvericin caused 37% mortality of *G. mellonella* after 12 days of incubation, with a median lethal time of approximately 6 days [54]. The LD_50_ of cordycepin was approximately 200 µg/larva 6 days post-injection [55]. In our experiments, a significant decrease in *G. mellonella* viability was observed at a concentration of 20 µg/larva (ca. 100 mg/kg) with an LT_50_ of ~7 days (Figure 8A). It was also shown for the first time that *Zophobas morio* larvae, a food insect used, for example, for feeding chickens [56], were also sensitive to injection with TeA at a dosage of 20 µg/larva (~140 mg/kg) with an LT_50_ of ~4 days (Figure 8B). These data demonstrate moderate levels of acute larvicidal activity of TeA comparable with entomotoxicity of beauvericin and cordycepin.

In oral assays, the addition of inactivated *Fusarium* spp. cultures to the feed of *G. mellonella* allowed the identification of the toxin-producing strains [57]. Diacetoxyscirpenol and neosolaniol isolated from *F. sambucinum* reduced the consumption of the spiked feed by *G. mellonella* ca. 50% compared with the untreated control on caterpillars at a concentration of 50 µg/g of feed [58]. Oosporein from *Beauveria brogniartii* was nontoxic for the insect at a concentration of 142 µg/g of feed [59]. The bioactive alkaloids harman and nonharman, found in the entomopathogenic fungus *Conidiobolus coronatus*, when given in a non-lethal concentration of 1.25 mg/g of feed caused a significant delay in the development of *G. mellonella* [60]. In our experiments, no dead *G. mellonella* larvae were observed at TeA concentrations of 2.5 mg/g of feed within 10 days of incubation. Moreover, TeA was not 100% lethal even at 25 mg/g (data not shown). However, at a concentration of 250 µg/g of feed, the growth of larval biomass was significantly inhibited (Figure 9). Low toxicity coupled with biomass loss may indicate a rather antifeedant effect of TeA for *G. mellonella*; however, this hypothesis needs further evidence. Nothing was previously known about the effect of mycotoxins on another food insect, *Acheta domesticus* [61]. When TeA was added to the cricket diet, significant insect mortality was observed at a concentration of 250 µg/g of feed with LT_50_ for approximately 7 days (Figure 10).

In the contact intestinal assays, TeA was found to have moderate aphicidal activity, with 40% mortality 24 h after treatment at a concentration of 2 mg/mL (Figure 11A), whereas beauvericin was considerably more toxic, causing 100% mortality at a lower concentration of 0.5 mg/mL. However, according to Ganassi et al. (2002) [62], beauvericin at the same concentration was less toxic to *S. graminum* (23% mortality). The differences in the aphicidal activity of beauvericin may be explained by the variability of *S. graminum* populations. As mentioned above, some other fungal toxins of the genus, for example, diversolonic esters and chloromonilinic acid B, are more toxic than TeA, causing 70–80% mortality of *S. graminum* at a concentration of 1 mg/mL [9,63]. The insecticide thiacloprid from the group of neonicotinoids was significantly more toxic to grass aphids, with an LD_50_ of approximately 210 µg/mL [64]. The sensitivity of aphids to seven organophosphorus insecticides varied within the LD_50_ range from 0.1 to 60 µg/mL, depending on the level of resistance of *S. graminum* populations [65].

*T. urticae* was less sensitive to TeA than *S. graminum*. However, significant mortality of slightly more than 10% was detected at 0.5 mg/mL after 24 h of exposure (Figure 11). Beauvericin showed noticeable acaricidal properties, with LD_50_ values from 0.66 to 33 µg/mL, depending on the sensitivity of the *T. urticae* population to the toxin [66]. The extract of the soil micromycete *Aspergillus melleus*, in which mellamide, ochratoxin C, nodulisporic acid, 7-oxocurvularin, and 6-(4′-hydroxy-2′-methyl phenoxy)-(−)-(3R)-mellein were detected, was significantly less toxic to the mites, with an LD_50_ of 10 mg/mL [67]. Thus, TeA demonstrated low activity against sucking insects in the contact intestinal assays. On other hand, evaluation of antifeedant and ovicidal effects of TeA on *S. graminum* and *T. urticae* appears interesting for further study.

Testing entomotoxic substances on insect and mammalian cells is helpful for the primary determination of the mechanisms of their action on arthropods [68]. Among 65 mycotoxins tested, TeA had relatively weak cytotoxicity to both insect and mammalian cells. It was shown that Sf9 cells are two times more sensitive to beauvericin than to TeA [69]. When tested on the porcine intestinal columnar epithelial cells (IPEC-J2 line), the cytotoxicity of TeA was 10 times lower than that of beauvericin [70]. However, in contrast to the latter, TeA caused rapid destruction of Sf9 cells at the minimum studied concentration of 15 µg/mL (Figure 12) and, therefore, may be of interest for mode of action studies.

### 4.3. Practical Implications

TeA, a derivative of tetramic acid, has gained great interest as a model molecule (toxin, pro-drug, and pro-pesticide) since (1) it is produced by a number of phytopathogenic fungi (*Alternaria* spp., *Magnaporthe oryzae*, and *Phoma sorghina*) [71]; (2) this toxin is one of the most common pollutants of food products, while its toxicity to invertebrates and vertebrates has been poorly understood [72,73]; (3) there are acaricides and insecticides (acetyl-CoA carboxylase inhibitors) from the tetramic acid group (for example, and spiropidion) that are effective against sucking arthropods [74]; (4) TeA, along with some other tetramic acid derivatives, is a promising natural herbicide [75], whereas its toxicity to beneficial insects (for example, pollinators, entomophages, and feed protein producers) has not been studied; and (5) methods of chemical synthesis of TeA and its derivatives with various useful properties have been developed [11,71,76,77]. However, the useful properties of direct use of TeA as a pesticide is still under question because of the restricted knowledge on its toxicity.

This study demonstrated moderate to low sensitivity of arthropods to TeA in various bioassays. Its entomotoxic activity against *G. mellonella*, *Z. morio*, and *Acheta domesticus* was shown for the first time. In natural materials, the TeA concentration does not commonly exceed the level of 100 ng/g and is clearly not entomotoxic. However, the maximum TeA content in food may vary greatly, reaching relatively high concentrations of up to 0.7 µg/g in grain products, up to 8 µg/g in vegetables, and up to 20 µg/g in spices [78,79]. Most likely, in cases of strongly moldy substrates or provisionally using TeA as an herbicide, the maximum concentrations can be sharply exceeded to affect sensitive arthropods. It makes sense to further study the spectrum of TeA entomotoxicity on harmful and useful insects (pollinators, entomophages, protein producers, etc.) and its action mechanisms on arthropods.

TeA has several molecular targets in the cells of various organisms. In plant cells, it inhibits the proton pump of the plasma membrane and photosynthesis [80,81], being one of the pathogenicity factors of phytopathogenic fungi that produce it [82]. Cell-free assays demonstrated TeA to be an antioxidant and a promising inhibitor of acetylcholinesterase and β-amyloid aggregation [83,84]. TeA is toxic to rodents and chickens, causing diarrhea, bleeding, and precancerous conditions. The toxin was presumed to negatively affect the digestive system of sensitive animals, but the exact mechanism of action of TeA is still unknown [73,85].

The absence of considerable behavior and developmental changes of the treated insects, lack of tremor, weak sensitivity of sucking phytophages, and clear antifeedant effect may indicate that TeA is not an inhibitor of lipid biosynthesis as spirotetramat is; instead, it is an intestinal toxin for sensitive insects such as *G. mellonella* [86]. The mode of action of this toxin can be evaluated further using insect model systems, for example, on *G. mellonella* larvae and Sf9 cells. This may partially solve the enigma of the mammal toxicity of TeA as well as promote this compound as a pro-pesticide [87].

## 5. Conclusions

The extracts of *A. tenuissima* MFP253011 obtained by various methods showed a wide range of biological activity, including entomotoxic properties. Analysis of their composition and bioactivity allowed us to reveal several known mycotoxins and unidentified compounds that may be involved in the entomotoxic activity of the extracts. PCA of the data on the biological activity and the chemical composition of the extracts predicted that TeA has larvicidal activity against *G. mellonella*. Indeed, for the first time, TeA was found to display moderate entomotoxic properties, which were manifested in injection and oral tests to this insect and several others (*Z. morio* and *A. domesticus*).

The action mechanisms of TeA can be evaluated further using insect model systems, for example, on *G. mellonella* larvae and Sf9 cells. Another interesting issue for future studies is to determine the indirect effects of TeA on arthropods: are the entomotoxic effects correlated with phytotoxicity, are the intestinal microbiota of arthropods suppressed, and does this toxin affect their humoral immunity and sensitivity to entomopathogens?

## Data Availability

All relevant data are included within the manuscript.
